# Microplate-Test for the Rapid Determination of Bacteriophage-Susceptibility of *Campylobacter* Isolates—Development and Validation

**DOI:** 10.1371/journal.pone.0053899

**Published:** 2013-01-17

**Authors:** Samuel Fischer, Sophie Kittler, Günter Klein, Gerhard Glünder

**Affiliations:** 1 Clinic for Poultry, University of Veterinary Medicine, Hannover, Lower Saxony, Germany; 2 Institute of Food Quality and Food Safety, University of Veterinary Medicine, Hannover, Lower Saxony, Germany; Charité, Campus Benjamin Franklin, Germany

## Abstract

A simple susceptibility test using 800 isolates of one *Campylobacter* strain with different degrees of susceptibility and four bacteriophages of the British phage typing scheme was developed and examined for its suitability. The test presented is economically cheaper and less time consuming than the conventional agar overlay plate assay and therefore enables the monitoring of changes in the susceptibility pattern during phage therapy under practical field conditions. The main objective of this study was to compare the simplified test with the conventional agar overlay plate assay. The conventional test describes for a population of *Campylobacter*: i. the rate of resistant isolates (0 plaques) and ii. the degree of susceptibility, also called relative efficiency of plating (EOP), for the remaining isolates. The simplified test divides the isolates into four susceptibility ranks, which are easily distinguishable to the naked eye. Ten *Campylobacter* isolates out of each rank were subjected to the conventional method for validation of the simplified test. Each resistance rank contained isolates showing certain degrees of susceptibility, reflecting decreasing susceptibility by an increase of the rank. Thus, the simplified test correlated well with the conventional method. Nevertheless, it can be suggested for a clear cut to summarise the first thee ranks as “high susceptible” and to mark out the fourth rank as reduced susceptible. Further test improvements will enable the monitoring of the degree of susceptibility and potentially also of resistance during phage therapy in the field. To ensure a long-lasting successful use of phage therapy, further studies on both the loss of susceptibility and the development of resistance of *Campylobacter* against phages combined with their impact on phage therapy will be necessary.

## Introduction

### *Campylobacter* as challenge

Human campylobacteriosis is presently the most frequent foodborne zoonosis in Germany and many other industrial nations. In most cases, the causative organisms are *Campylobacter* (*C.*) *jejuni* and *C. coli*, which are mainly transmitted by poultry meat [1], [2], [3], [4], [5], [6], [7]. A reduction of intestinal colonisation of broilers would lead to a considerable decline of human campylobacteriosis [8]. A risk assessment by the use of a mathematic model shows that a reduction of 2 lg of *Campylobacter* counts on broiler carcasses leads to a 30-fold decline in human campylobacteriosis caused by chicken meals [9].

### Phages as approach

An investigation by Atterbury *et al.* [10] showed that phages are able to reduce *Campylobacter* counts by up to 1.3 lg on the surface of experimentally contaminated broiler skins. In several in-vivo studies on the reduction of *Campylobacters* by the use of phages a loss of *Campylobacter* counts to 2 lg as the mean were found, the highest reduction detected being 5 lg CFU [11], [12], [13], [14]. Thus, phages are most probably a supplementing tool for the production of safe food [15]. The usage of phages for farm animals is of particular interest to reduce the administration of antibiotics. Antibiotic medication leads only to a temporary reduction, but not to an elimination of *Campylobacter* [16] and attracts long-lasting public criticism regarding increasing bacterial resistances.

### Resistance and loss of susceptibility as obstacle

Even when phages were used to reduce *Campylobacter* counts in the intestine of poultry, phage resistant *Campylobacter* isolates were discovered. Thereby, resistance rates from 2% [12] up to 13% [11] were found. The phage therapy of the experimentally *Campylobacter* contaminated broiler skin subsequently did not result in the appearance of resistant isolates. “However, in the absence of replication these studies do not rule out the generation of new mutational events selected post treatment” [10].

The impact of resistant *Campylobacter* isolates on the following phage therapy was further examined. For this, the colonisation capability of resistant isolates *in vivo* and also the preservation of resistance *in vitro* and *in vivo* were investigated. Loc Carrillo *et al.* [13] found a significantly reduced capability of a phage resistant isolate to colonise the broiler intestine compared to the susceptible original strain, whereas Carvalho *et al.* [11] (2010) could not confirm such a difference in comparable studies. The findings of investigations concerning the preservation of resistance during a further intestinal passage differ highly. The percentage of isolates, which lost their resistant phenotype, was specified by the mentioned authors as 54% up to 97%. Loc Carillo *et al.* [13] found that *in vitro* produced resistant isolates keep their resistance over a sample period of about one hundred generations, represented by five subcultivations on horse blood agar.

Moreover, increasing host cell restriction acquired after phage treatment could become too great to be overcome and is therefore a critical point for phage therapy.

### Usage and meaning of the term “resistant”

To date, isolates showing no plaques when examined with a phage strain using the surface droplet technique were regarded as resistant. To determine the degree of resistance, serial dilutions of phage suspension were used [13] and “the phage titers obtained from lawns of the recovered *Campylobacters* were compared with titers determined with the initial strain” [10].

The term “resistant” was also used in line with the description of lytic profiles, which are defined as the ability of elected phages to infect a *Campylobacter* strain [17]. In the case of lytic profiles, low susceptible strains, showing less than 20 PFU post application of 10 µl Routine Test Dilution (RTD) to a lawn of the particular strain were denoted as resistant or nonsusceptible, according to Frost *et al.* [18]. The RTD is a phage suspension at a concentration, which produces semiconfluent lysis on a bacterial lawn of the reference strain after application of 10 µl phage suspension [19]. El Shibiny *et al.* [12] suppose that these low susceptible strains could not be successfully suppressed by phage therapy with the particular phage. Grajewski *et al.* [20] even categorise isolates, showing less than 50 PFU when examined with a RTD, as a negative result. Basis for this classification was a U-shaped frequency scale of isolates between 0 PFU and 100 PFU with a minimal turning point at 50 PFU. It would be skilfully, to name all these isolates as “reduced or insufficient susceptible” instead of “resistant”, to differentiate clearly between inherent and acquired causes.

### Development of a Microplate-Test for susceptibility analysis

**Figure 1:**
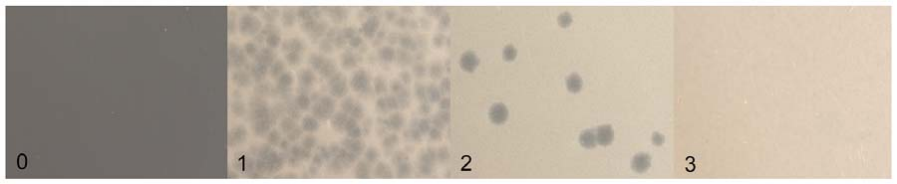
Examples of the four susceptibility ranks in the Microplate-Test. The pictures show a close up view of the four susceptibility ranks (from left to right): rank 0 = confluent lysis, high susceptible; rank 1 = semiconfluent lysis, susceptible; rank 2 = single plaques, reduced susceptible; rank 3 = no plaques, low susceptible and resistant.

Better understanding of the development of reduced susceptibility or resistance will be a decisive factor regarding the successful long-term use of phage therapy for quantitative reduction of *Campylobacter* on food and furthermore for the reduction of foodborne human campylobacteriosis. Studies about the susceptibility of various *Campylobacter* colonies, reisolated in further experimental investigations in poultry, will be necessary to understand effects and interactions of reduced susceptible and resistant isolates in the poultry intestine. The objective of our work was to develop a susceptibility and resistance test, which i) can be accomplished and read in a minimal amount of time and with a minimal amount of work and material costs and ii) manages to divide *Campylobacter* isolates into four susceptibility ranks. Four ranks were chosen in a quest for early detection of minimal loss of susceptibility on the one hand and for determination of low susceptible and resistant isolates on the other hand. For subdivision of these ranks we decided to take a key, which is easily visible to the naked eye (Table 1, Figure 1).

**Table 1:** Interpretation key of Microplate-Test.

key No.	degree of sensitivity | resistance	description
0	high susceptible	confluent lysis
1	susceptible	semiconfluent lysis
2	reduced susceptible	single plaques
3	low susceptible and resistant	no plaques

## Materials and Methods

### Ethical statement on bird experiment

This study was carried out in strict accordance with the recommendations in the Guide for the Care and Use of Laboratory Animals of the National Institutes of Health. The use of animals in this study was approved by the animal welfare officer of the University of Veterinary Medicine Hannover, whose tasks include the scrutiny of animal welfare, ethics and handling, and then announced to the Lower Saxony State Office for Consumer Protection and Food Safety according to §8a(1,2) of the German Animal Health and Welfare Act. The Lower Saxony State Office for Consumer Protection and Food Safety approved the work on this study under permit number 33.9-42502-05-11A153. The study was notifiable but not subject to approval according to § 8(7) Nr. 2 of the German Animal Health and Welfare Act. The accomplishment of the experiment was supervised by a competent person according to the §9 of the German Animal Health and Welfare Act to ensure the compliance of §9 and §9a of the German Animal Health and Welfare Act and all efforts were made to minimize suffering.

### *Campylobacter*

In 2006 the *C. jejuni* strain 1474-06 was isolated in an abattoir from poultry pectorals. This strain is representative for many field strains [21] and susceptible to all phages used in the United Kingdom Typing Scheme except phage 12 (NCTC12677) [22]. This strain was used for the development and the proving of the Microplate-Test.

*Campylobacter* strains, used for propagating phage strains and determining phage concentration are listed in Table 2.

**Table 2:** Phage strains and their appropriate *Campylobacter* strains.

phage strains		*Campylobacter* strains (NCTC^1^-No.)	
phage-No.	NCTC^1^-No.	propagation	determination of concentration
φ1	12673	12661	12662
φ2	12674	12661	12662
φ5	12678	12664	12662
φ13	12672	12660	12662

1 National Collection of Type Cultures.

All required *Campylobacter* strains were cultured and stored in aliquots at −70°C to serve as a master seed before studies commenced.

*Campylobacter* was generally cultivated under microaerobic conditions (Oxoid Gas Pak System, Campygen) at 42±0.5°C.

*Campylobacter* 1474-06 for inoculation of broilers was propagated in Standard 1 nutrient broth (Merck) for 48 h. Reisolation was performed on Karmali Agar (Oxoid). For this, serial dilutions of 1 g of caecal contents per broiler were made in phosphate buffered saline (8 g NaCl, 2 g KH_2_PO_4_, 2.9 g Na_2_HPO_4_, H_2_O ad 1 l, adjusted to pH 7.4 with HCl/NaOH). 100 µl from each dilution was plated on Karmali Agar twice in order to obtain at least ten single colonies from each bird for the Microplate-Test.

To use *Campylobacters* for propagating or determining phage concentrations as well as for the resistance test, a single colony of a working culture was plated on Mueller-Hinton-Agar and incubated for 16 h. Then bacteria were harvested with a sterile cotton swab, dispensed in 10 mmol MgSO_4_ solution and adjusted to the Mc-Farland-Standard (McFSt.) needed for the particular application in a densimat (Biomerieux SA France, IDN 013615).

### Bacteriophage propagation

The phages 1, 2, 5 and 13 (Table 2) from the British phage typing scheme were used for this study.

The procedure for propagating phages and determining their concentration was conducted after modifying the method of Hansen *et al.* [23].

For phage propagation a suspension of the particular *Campylobacter* strain (Table 2) was adjusted to McFSt. 3. NZCYM-overlay-agar (NZCYM broth 22 g/l, Roth X974.1, Agar-agar 7 g/l, Roth 2266.3) was liquefied in tubes and kept molten at 48±0.5°C in a block heater (Roth, Rotalibo-Block-Heater H250). 100 µl of the *Campylobacter* suspension and 100 µl of the phage-suspension were added to 5 ml NZCYM-overlay-agar, mixed thoroughly and poured into a petri dish containing 20 ml NZCYM-base (NZCYM-broth 22 g/l, Agaragar 15 g/l). After the overlay had solidified, plates were inverted and incubated at 42±0.5°C for 24 hours (phages 1, 2, 5) or 48 hours in the case of phage 13. Subsequently, 5 ml SM-Buffer (5.8 g NaCl, 2.0 g MgSO_4_×7H_2_O, 50 ml 1M Tris (Sigma) pH 7.5, 5 ml 2% gelatine, Aqua dest. ad 1000 ml) were added to plates showing semiconfluent up to clear lysis. Plates were swayed on an orbital shaker at 100 rpm overnight at 4°C to allow the phages to pass into the buffer. Chloroform (Sigma-Aldrich, 366919) was added to the phage containing recovered SM-buffer at a concentration of 5% (v/v), which was then shaken at 300 rpm for 15 min and subsequently centrifuged at 13000× g for 20 min. The supernatant was filtrated through a 0.22 µm filter (Roth, P668.1) to eliminate bacterial compounds. The obtained phage suspension was stored at 4°C.

### Determining phage concentration

For determining phage concentration a log_10_ dilution series of the respective phage was prepared and a suspension of *C. jejuni* strain NCTC 12662 was adjusted to McFSt. 5. Aliquots of 100 µl of the phage and *Campylobacter* suspensions were added to 5 ml NZCYM-Overlay liquefied at 48±0.5°C. Thus, two tubes of each dilution were prepared and then poured onto plates containing 20 ml NZCYM-Base. The plates were incubated under microaerobic conditions for 16 h after the overlay had solidified. For determining the phage concentration in the suspension the weighted arithmetic average of the counted plaques was calculated [24].

### Production and preparation of *Campylobacter* isolates resistant or with different grades of susceptibility to phage infection

Altogether, 80 broilers, tested negative for *Campylobacter* colonisation, were inoculated into the crop with 0.5 ml nutrient broth containing 10^4^ CFU *C. jejuni* strain 1474-06 at day 6 of life and kept in separate isolation units to avoid any environmental cross contamination. At day 9 of life 1 ml of SM-buffer supplemented with 33% (w/v) CaCO3 (Roth, 6230.1) containing a cocktail 10^7.5^ PFU of phages 1, 2, 5 and 13 each was administered directly into the crop of the broilers. To obtain *Campylobacter* isolates with different grades of resistance, 8 different days (day 1,3,7,14,21,28,35 and 42) after phage application were chosen for re-isolation of *Campylobacter* from caecal contents of the broilers. Ten single colonies from each broiler were chosen and tested for resistance. Identifying these isolates as belonging to the species *C.* jejuni was confirmed by testing one colony per broiler by PCR. The PCR was performed as the hippuricase PCR, described by Marshall *et al.* [25]. Thus, 800 *Campylobacter* colonies from the inoculated broilers and a further 100 colonies from the original *Campylobacter* suspension before inoculation of the trial birds were available for the tests for the acquired resistance. These 900 isolates were necessary to gain a sufficient number of resistant isolates for evaluating the Microplate-Test, based on a resistance rate of 2% [12].

### Microplate-Test

**Figure 2:**
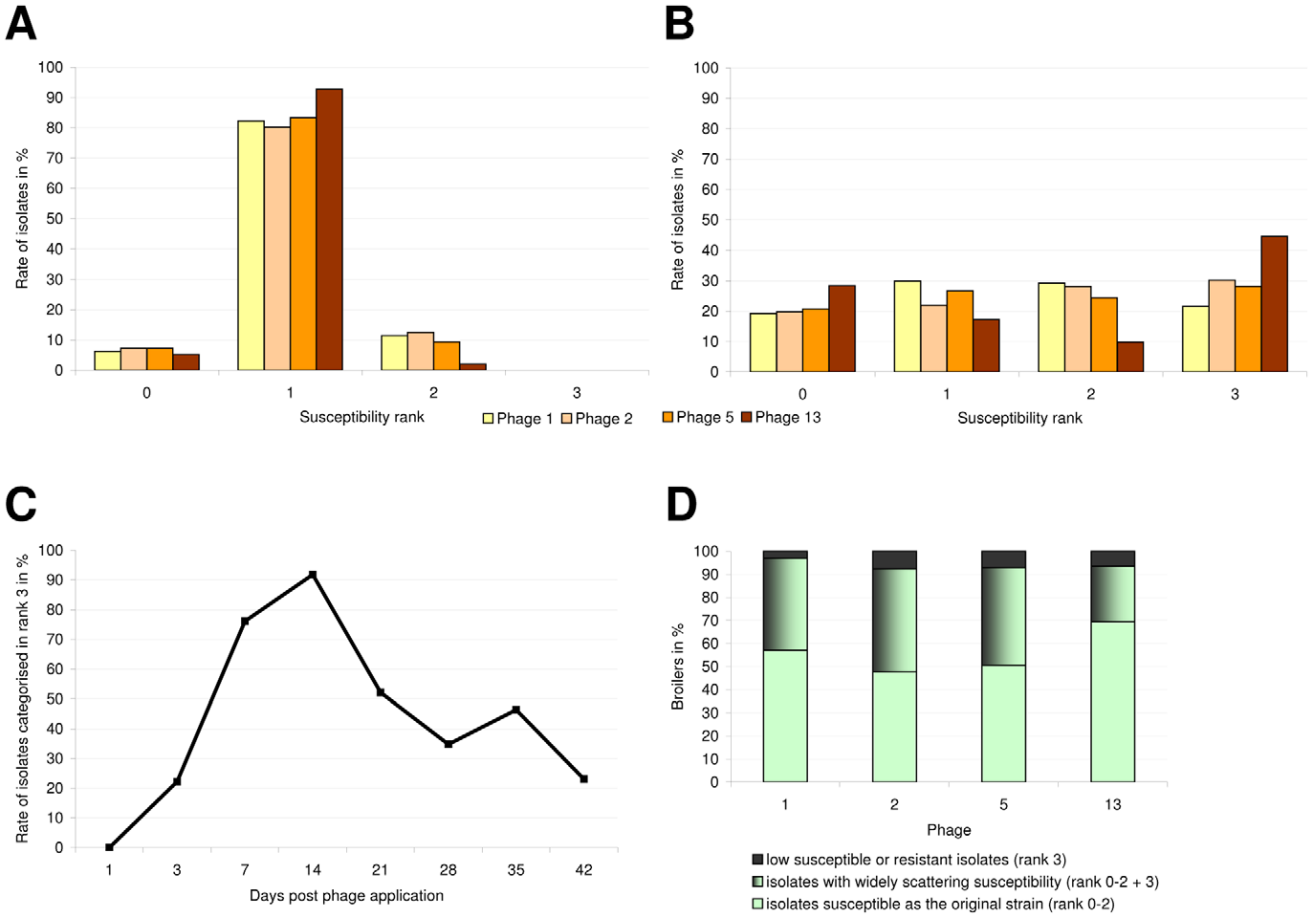
Phage-susceptibility of *C. jejuni* strain 1474-06. Part A and B show the rate (%) of *Campylobacter* isolates belonging to the respective rank before and after phage treatment. Part A shows the susceptibility pattern of *C. jejuni* against four different phages without phage treatment. Hundred isolates of the original strain were examined in the Microplate-Test. Part B shows the susceptibility pattern of reisolated *C. jejuni* against four different phages after phage treatment *in vivo* in the intestinal tract of broilers. Eight hundred isolates were examined in the Microplate-Test. Part C shows the development of susceptibility of *C. jejuni in vivo* after phage treatment over the course of time. The graph shows the rate of isolates in rank 3 representing low susceptible and resistant isolates for the phage cocktail on each day of sampling. Part D shows the susceptibility of *Campylobacter* isolates originating from one chicken. The bars show the percentage of chicken, harbouring isolates with i) only similar susceptibility as the original strain ii) broad scattering susceptibility and iii) only low susceptibility or resistance.

The susceptibility test was performed in a 6×8 well microplate (Sigma-Aldrich, CLS3548). Every *Campylobacter* isolate was tested against all four phages per one individual well and a further well was used for growth control of *Campylobacter*. Appropriate to RTD, the phage-containing suspensions were prepared containing 10^6^ PFU/ml of phages 1, 2, 5 or 10^7^ PFU/ml of phage 13, respectively (these concentrations were chosen from preliminary screenings using colonies from the original strain in the Microplate-Test with different phage concentrations, data not shown). The RTD, producing semiconfluent lysis was chosen in order to allow the detection of an increase and decrease of lysis in the Microplate-Test. Afterwards the 100 colonies of the original strain were tested in the Microplate-Test in order to confirm a constant output (Figure 2A). The phage suspensions were stored at 4°C until use. From every phage suspension 10 µl were transferred into the appropriate wells and 10 µl of test *Campylobacter* suspension, adjusted to McFSt. 5, were added. Subsequently, 0.5 ml of modified NZCYM-overlay composed of 22.0 g NZCYM-powder, 3.5 g Agaragar, 3.5 g Low-melting-agar (Promega Corporation, V2111) per 1000 ml were filled into each well. The plates were incubated under microaerobic conditions after solidification at 42±0.5°C for 18 h.

The interpretation of the test ranging from totally susceptible to resistant was conducted according to the key described in Table 1. The susceptibility ranks are shown in Figure 1.

### Comparison of Microplate-Test and method of conventional resistance tests

In the conventional resistance test isolates are regarded as resistant when showing 0 plaques. The detection threshold was up to 5 PFU/ml for isolates judged as resistant in the conventional test. Isolates showing one or more plaques were regarded as partially susceptible and the degree of susceptibility was determined as the relation of generated plaques to applied PFU, which is called relative efficiency of plating (EOP) by Kutter [26]. Kutter describes that because of host factors, the relative EOP, as the titer of the phage on a given bacterial cell line compared to the maximum titer observed, may vary considerably. Sorensen *et al.* [27] instead use the term efficiency of plaquing (EOP). They examined the susceptibility of a *C. jejuni* strain to bacteriophages by using their plaque assay protocol. This Protocol describes the bacteriophage titration and was used to determine the EOP in percent by dividing the PFU/ml on the test strain by the PFU/ml on the control strain multiplied by 100.

In summary conventional plaque assays are used to determine the resistance (0 Plaques) or degree of susceptibility as EOP of particular *Campylobacter* isolates [10]. EOP is determined on a metric scale.

In the Microplate-Test four ranks of susceptibility were differentiated for each of the four phages examined. The ranks are arranged on an ordinal scale. For all four susceptibility ranks each phage was subjected to the conventional test against at least 10 *Campylobacter* isolates. The relative EOP of the chosen *Campylobacter* strains was tested as described above under determination of phage concentration. Specifically, the concentration of each of the four phages was adjusted to 10^8^ PFU/ml in SM-buffer with *C. jejuni* strain NCTC12662 as standard. The dilution series of these standards were tested against the selected *Campylobacter* isolates and the plaques were counted.

The relative EOP allows a comparison of the four phages: the most susceptible isolate showing the highest counts of plaques was defined as 100% susceptible. Lower counts of plaques with other *Campylobacter* isolates were correlated to the highest counts with the susceptible strain and the relative susceptibility was defined in percent as described by Kutter [26].

The Microplate-Test uses one tenth of the quantities of phage suspension, bacteria suspension and overlay medium compared to the conventional method, but only one phage concentration (10^6^ or 10^7^) instead of phage dilution series (10^0^ to 10^8^). Therefore the added ratio of phages to bacteria, representing multiplicity of infection (MOI) input, in the Microplate-Test is the same as for the 10^6^ or 10^7^ dilutions in the conventional assay.

## Results

### Microplate-Test

#### Susceptibility of Campylobacter before and after phage treatment (Figure 2A and 2B)

First, 100 isolates from the original strain were used to confirm the correct phage concentration for each phage, needed to produce semiconfluent lysis in the Microplate-Test (Figure 2A). Two isolates were not appraisable. More than 80% were categorised in rank 1, which is defined as showing semiconfluent lysis. Less than 13% were categorised in rank 0 or rank 2. No Isolate was categorised in rank 3.

Subsequently, 800 *Campylobacter* isolates from strain 1474-06 colonised chickens were examined for their susceptibility against four phages in the Microplate-Test and classified into four ranks with decreasing susceptibility from ranks 0 to 3. *Campylobacter* isolates could be related in nearly equal quantities to each rank, as shown in Figure 2B. Only loss of susceptibility for phage 13 was more pronounced, so that 45% of the isolates could be categorised in rank 3.

#### Development of susceptibility over the course of time (Figure 2C)

One day after phage application, there was no change in phage susceptibility of the *Campylobacter* test population. Over the next two weeks phage susceptibility decreased and subsequent increased until the end of the investigation period.

#### Susceptibility of Campylobacter isolates originating from one chicken (Figure 2D)

For examination of the range of susceptibility of *Campylobacter* isolates origination from one chicken we collected i) isolates from rank 0–2 in one group, representing the range of susceptibility of the original strain, ii) isolates of rank 3 in the second group, representing low susceptible and resistant isolates. 55%–60% of the broilers harboured isolates from one group in the case of phages 1, 2, 5 and 76% of the chickens were colonised by similar susceptible isolates for phage 13. 24%–45% of the birds harboured isolates with a broader range of susceptibility.

### Explanatory power of Microplate-Test

**Figure 3:**
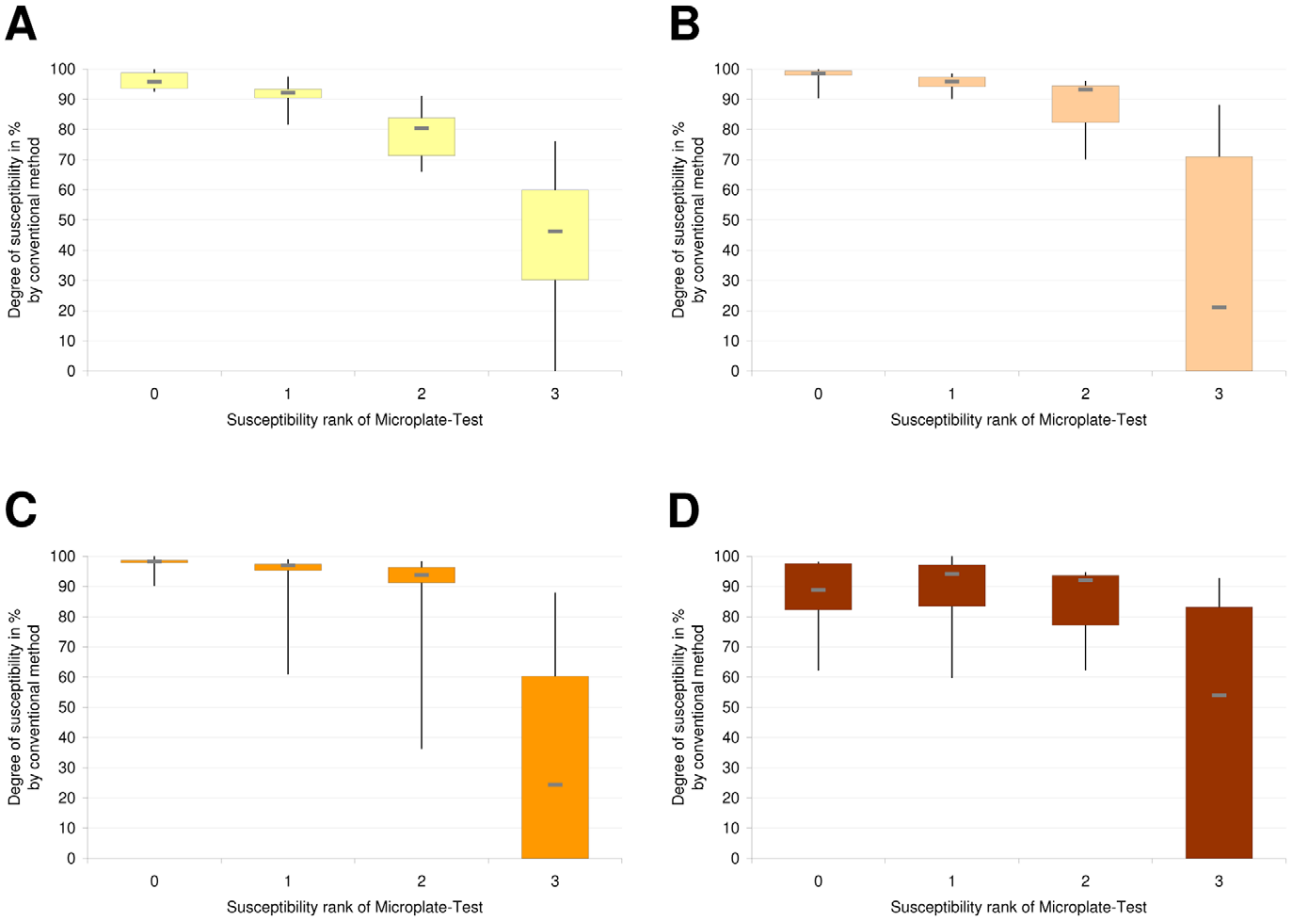
Comparison of the Microplate-Test and the conventional method. Part A shows the degree of susceptibility for infection by phage 1 of isolates from the four susceptibility ranks. (a,b,c,d: significance, p<0.005). Part B shows the degree of susceptibility for infection by phage 2 of Isolates from the four susceptibility ranks. (a,b,c,d: significance, p<0.02). Part C shows the degree of susceptibility for infection by phage 5 of Isolates from the four susceptibility ranks. (a,b,c: significance, p<0.003). Part D shows the degree of susceptibility for infection by phage 13 of Isolates from the four susceptibility ranks. (a,b: significance, p<0.02).

For comparison of the Microplate-Test and the conventional method of susceptibility and resistance determination, at least 10 *Campylobacter* isolates from each susceptibility rank were subjected to the conventional method. The degree of susceptibility for each *Campylobacter* isolate was noted as the number of plaques produced by a defined phage-suspension on these isolates (Table 3). Each susceptibility rank of the Microplate-Test corresponded to a defined amount of degrees of susceptibility by conventional examination (Figures 3A–D). Rank 0–2 contain high susceptible isolates, rank 3 contains a broad range of less susceptible and resistant isolates.

**Table 3:** Comparison of Microplate-Test and conventional method.

Microplate-Test	conventional method			
susceptibility rank	degree of susceptibility/EOP^1^ in PFU/ml (lg)			
	Phage 1	Phage 2	Phage 5	Phage 13
0	7.8–8.4	7.1–7.9	7.4–8.2	4.0–6.4
1	6.8–8.1	7.1–7.8	5.0–8.1	3.9–6.5
2	5.5–7.6	5.5–7.6	3.0–8.1	4.1–6.2
3	0–6.3	0–6.9	0–7.2	0–6.0

1 EOP = Efficiency of Plating.

Phages 1, 2 and 5 showed similar characteristics in the Microplate-Test (Figures 3A–C). In nearly all cases the susceptibility differed significantly between the four susceptibility ranks. Only in case of phage 5 (Figure 3C) the number of *Campylobacter* isolates examined did not suffice to reach a statistically significant difference between ranks 0 and 1 or rather ranks 1 and 2. Ranks 0 to 2 differentiated small losses of susceptibility in fine steps, whereas rank 3 included less susceptible up to resistant isolates (no plaque by conventional examination) for the three phages mentioned. In contrast, the Microplate-Test did not differentiate between ranks 0 to 2 in the case of phage 13, but differentiated significantly between high susceptible (ranks 0–2) and less susceptible plus resistant (rank 3) isolates (Figure 3D).

The Microplate-Test detected resistant isolates with a sensitivity of 100% as belonging to rank 3 for all phages. For the appropriate *Campylobacter*-phage combinations the specificity of Microplate-Test for the detection of resistant isolates in rank 3 is 17%, 30%, 50% and 36% in the case of phages 1, 2, 5 and 13, respectively. Using the threshold of 20 plaques in the conventional test, mentioned in previous studies as the threshold of effectivity in lytic profile examinations [12], the specificity therefore increases to 50% or 60% in the case of phages 1 and 2.

## Discussion

### Results of the Microplate-Test

When examining the 800 phage treated *Campylobacter* isolates on average 31% (22%–45%; Figure 2B) were classified into rank 3. In consideration of the specificity for detecting resistant isolates in rank 3, we can assume a resistance rate of 4, 9, 14 and 16% for phage 1, 2, 5 and 13 respectively. Therefore, the rate of resistance found in this study is comparable to those rates of resistance published for comparable examinations where 13% was the highest result [11].

Until now, there have been only few examinations published focussing on the emergence of resistance of *Campylobacter* against phages *in vivo* [10], [11], [12], [13], [28]. Differences in observed rates of resistance can be explained by the application of different phage-strains to certain *Campylobacter* strains, by differences in dose and duration of treatment or time of sampling after treatment. If such high differences in rates of resistance as observed in our study occur against phages, this would be a relevant clue for the appropriate selection of phages for phage therapy. To build a phage library, phages having little risk of provoking resistance should therefore be chosen.

### Interpretation of the Results of the Microplate-Test

We considered the reliability of the Microplate test by including a variety of four different phages. Furthermore, we have chosen carefully the *Campylobacter* test strain, which derived from a collection of well characterised field strains and is representative for the field strains. The reliability of the test would be better if a further test population based on one or two *Campylobacter* strains had been tested.

Plaque assays detect plaque forming activity. Plaque forming activity allows the conclusion “susceptible”, whereas no plaque forming activity leaves open different ways of interpretation like resistance or plaque formation failure, which are discussed in detail by Abedon and Yin [29]. Therefore assays based on plaque forming activity generally must be interpreted with the provision that every plaque assay possibly has a margin for error in detecting phage susceptibility of bacteria.

In addition, including the results of further investigations concerning the mechanisms of host cell restriction and resistance as well as their impact on phage selection for suitable therapy will be necessary [28], [30], [31], [32], [33], [34], [35], [36] for the correct interpretation of the test results.

### Validation of the Microplate-Test

#### Results of Validation

The results obtained with the Microplate-Test were validated by the conventional method. This evaluation resulted in comparable findings for phages 1, 2 and 5. For these phages, all susceptibility ranks were distinguishable and represented a decreasing susceptibility of *Campylobacter* isolates from ranks 0 to 3. Ranks 0, 1 and 2 are located in the high susceptible area near each other. Thus, the Microplate-Test already recognises a minimal decrease of susceptibility indicating that the test is suitable for detecting early emergence of host cell restriction.

In the case of phage 13, no difference could be detected between ranks 0 to 2. This indicates the need to recheck the correct application of the Microplate-Test for each new phage. For phage 13, the Microplate-Test differentiated only between high and low susceptible. In such cases, ranks 0 to 2 have to be combined.

#### The correct MOI

Rank 3 contains isolates with a broad range of degrees of susceptibility and resistant isolates (Figures 3A–D, Table 3). The accurate part of resistant isolates (0 plaques) is only detectable by the conventional method. The absence of plaques in the Microplate-Test is not equivalent to the absence of plaques in the conventional method. This might stem from different MOI actual, representing the infection ratio of phages to bacteria, and therefore different time scales for bacterial growth and phage propagation. In order to achieve the correct MOI actual, correct phage and bacteria numbers (MOI input), sufficient time of bacterial exposure to phages and sufficient phage penetration to bacteria are required [37]. The only variable in a susceptibility test system should be the phage infectivity for bacteria or vice versa the host cell restriction, resulting in different EOP. In the Microplate-Test, we took the same proportion of phages, bacteria and overlay medium as used for conventional agar overlay plate assay and applied it on multi-well cell culture plates instead of petri-dishes in order to assure the stability of the other plaque formation influencing factors. Therefore, MOI input, describing the added ratio of phages to bacteria is the same for both tests. Nevertheless, MOI actual, describing the ratio of adsorbed phages to bacteria, could be different for the Microplate-Test, compared to the conventional method, as a result of changed reaction and framework conditions, especially different time of bacterial exposure to phages until addition of overlay medium. This could cause failures in examinations of *Campylobacter* isolates in the Microplate-Test. One argument against this theory is that isolates from the original strain before phage treatment, which have a well defined constant susceptibility, showed high consistently semiconfluent lysis in the Microplate test, indicating consistent infecting ratios in these examinations.

### Benefit of the Microplate-Test

The Microplate-Test allows in future partial automatisation for production and reading of a phage susceptibility test for *Campylobacter*. Compared to the conventional method, the Microplate-Test allows simultaneous examination of a fivefold to tenfold number of isolates, depending on the degree of automatisation.

## Conclusions

Based on the good correlation of the examined Microplate-Test and the conventional method the new test can be used for monitoring the development of phage susceptibility of treated *Campylobacter* populations during phage therapy experiments in research and later on in field use.

Thereby, the simple as well as time and cost effective application may allow a high number of examinations. In future, a sufficient/not sufficient susceptibility test may be of more relevance, i.e. combining the four ranks of susceptibility in the Microplate-Test to make two, considering the actual knowledge about inter-relations of successful phage therapy and phage susceptibility of bacteria strains in the field.
